# High patient satisfaction with Customized Total Knee Arthroplasty at five year follow-up

**DOI:** 10.1007/s00264-024-06325-y

**Published:** 2024-09-25

**Authors:** Philipp Schippers, Felix Wunderlich, Yama Afghanyar, Victoria Buschmann, Thomas Klonschinski, Philipp Drees, Lukas Eckhard

**Affiliations:** 1 Department of Orthopedics and Traumatology, University Medical Center of the Johannes Gutenberg University Mainz, Mainz, Germany; 2 Praxis für Gelenkchirurgie Bad Kreuznach, 55543 Bad Kreuznach, Germany

**Keywords:** Customized, individually made, Total knee arthroplasty, Conformis

## Abstract

**Purpose:**

Despite numerous studies demonstrating promising short-term outcomes of Total Knee Arthroplasty (TKA) with Customized Individually Made (CIM) implants, there is a significant lack of data on their mid-term effectiveness. Given the increasing number of TKAs performed annually, the rising demand for CIM implants, and the associated burden of revision surgeries, understanding the mid-term performance of CIM implants is crucial. Therefore, this study aims to report on the mid-term (minimum 5 years) outcomes of TKA using a CIM implant.

**Methods:**

This retrospective cohort study included a consecutive series of 116 patients who received the ConforMIS^®^ iTotal CR implant between 2015 and 2018. Inclusion criteria were end-stage knee osteoarthritis with coronal deformities below 10° and absence of ligamentous instability. Exclusion criteria included simultaneous bilateral TKA. Patients were followed up at a minimum of five years post-surgery. They completed a questionnaire reporting on satisfaction, pain levels using the Visual Analogue Scale (VAS), current weight, the Oxford Knee Score (OKS), and the Forgotten Joint Score for the knee (FJS-knee). Statistical analysis included descriptive statistics for demographic and clinical variables, and outcomes were reported as means with ranges.

**Results:**

The mean follow-up duration was 5.9 ± 0.8 years (range 5–7.4 years). 90% of patients stated they would undergo the same operation again, and 93% were either satisfied or very satisfied. The mean VAS for pain at rest was 2 ± 1.5 (range 0–6) and during exercise was 3 ± 2 (range 0–8). 58 patients (53%) managed to lose weight. The mean OKS was 41 ± 9 points (range 15–48), and the mean FJS-knee was 67 ± 23 points (range 4–100). No severe complications occurred.

**Conclusion:**

CIM TKA using the ConforMIS^®^ iTotal CR implant can achieve excellent results with 93% of patients being satisfied or very satisfied at mid-term follow-up of five years. Prospective, randomized, and patient-blinded trials comparing off-the-shelf (OTS) TKAs with CIM implants are necessary to evaluate whether these implants are superior or not.

## Introduction

Several causes for the failure of TKA have been discussed in the literature. Apart from patient-related factors, many failures can be attributed to limited abilities in restoring patient anatomy due to standardized sizes of “Off The Shelf” (OTS) implants. Their sizes are based on anthropometric measurements of a standard population [1]. However, recent studies have highlighted a significant variance in the anatomy among humans [2–4]. This results in a challenge to treat patients with less conventional anatomy. Surgeons are therefore often forced to compromise between various intra-operative challenges. One significant issue is balancing the flexion space when the anteroposterior (AP) and mediolateral (ML) dimensions do not match, which can lead to difficulties in achieving proper ligament tension and stability [5]. Additionally, changes in the trochlear valgus angle, especially when applying certain alignment strategies, can generate significant issues, such as maltracking and patellofemoral complications [6]. While tibial component overhang, particularly on the lateral side, is known to cause popliteal irritation [7], femoral component overhang is less consistently problematic, which is why narrower components have not gained the expected popularity [8]. In fact, anatomic variances make optimal implant fitting on the tibial side almost impossible [2]. Furthermore, on the tibial plateau, a cut-off between maximizing fit and malrotation is often reported [9, 10].

To address the challenges mentioned above and to more accurately restore patient anatomy and kinematics, Customized and Individually Made (CIM) implants were introduced. With preoperative CT, 3D models of the patient’s joint are rendered and used to create custom implants tailored to the patient’s specific bony and ligamentous anatomy and the desired alignment strategy [11]. It is hypothesized in the literature that personalized anatomic design leads to improved implant fit with consequent enhancement in stability and function to reduce surgical revisions and optimize Patient-Reported Outcome Measures (PROMs) [12–15].

Many studies have shown promising short-term outcomes [16–18], yet mid-term results for CIM implants are still scarce. Hence, the present study aimed to report mid-term outcomes after treatment of OA of the knee with CIM implants using the ConforMIS^®^ iTotal CR system.

## Methods

### Ethical approval and GCP statement

This study was conducted in accordance with the principles of the Declaration of Helsinki and Good Clinical Practice (GCP) guidelines. Ethical approval was obtained from the Institutional Review Board (IRB) of the Ethics committee of Rhineland-Palatinate, Germany (Approval Number: 2018–13930).

### Study design and participants

**Fig. 1:**
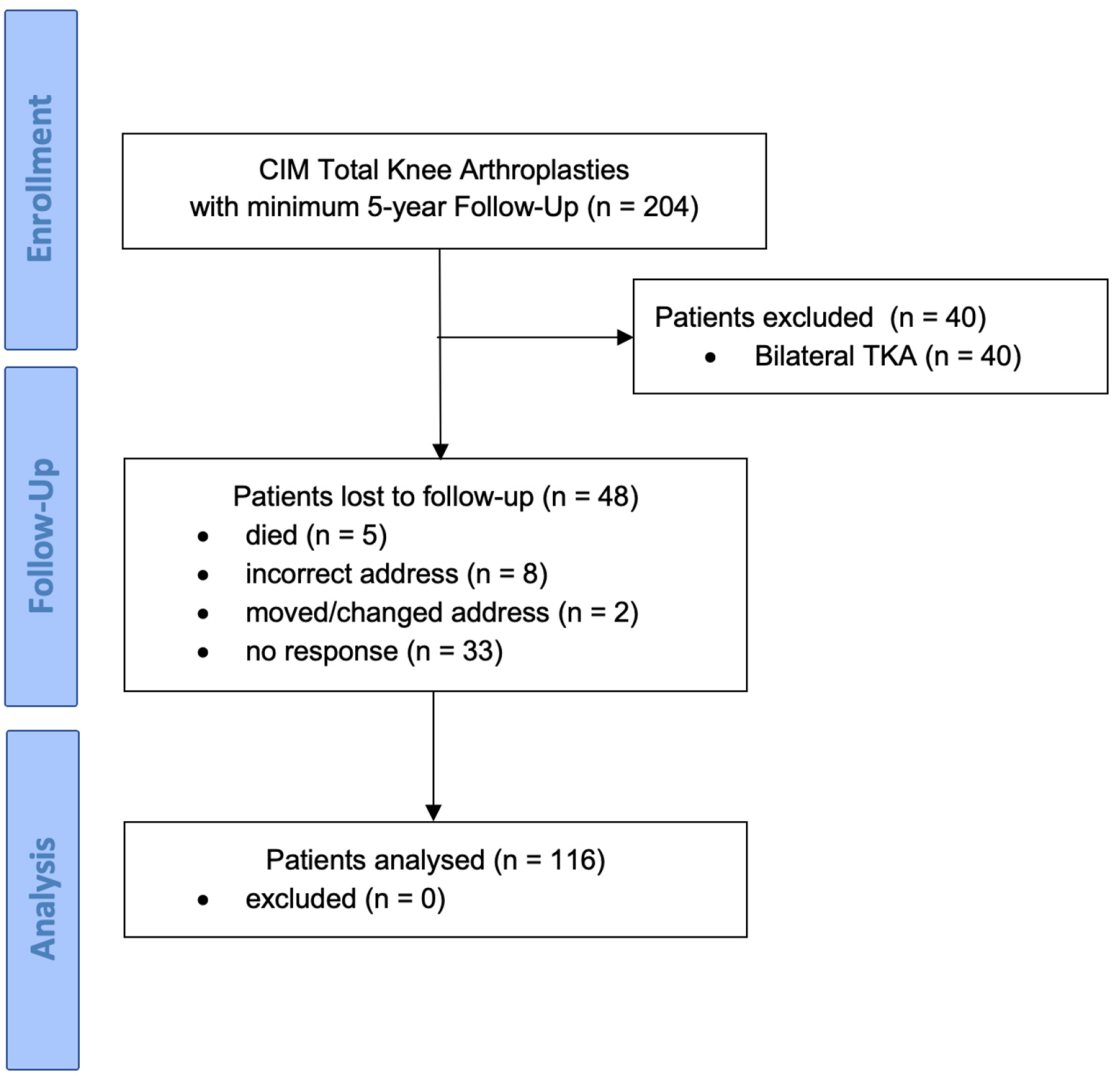
Consort flow chart

Between 2015 and 2018, 204 patients received a TKA using the ConforMIS iTotal CR implant. Patients with end-stage knee osteoarthritis with coronal deformities below 10° and absence of ligamentous instability were eligible for surgery with the evaluated implant. Patients who received simultaneous bilateral TKA (*n* = 40) were excluded. Patients were followed prospectively and routinely received a letter for self-reporting of their outcome five years after the surgery. Among the remaining 164 patients, there were 79 procedures performed on the right side and 85 on the left side; patient demographics are shown in Table 1. 29.3% of the patients were lost to follow-up; thus, 116 patients were analyzed (Fig. 1).

**Table 1:** Patient demographics

Sex distribution	86 woman, 78 men
Mean age (range)	65.7 years (range 33–87)
Mean BMI (range)	29.2 kg/m2 (range, 18–51)

### Surgical technique and alignment strategy

**Fig. 2:**
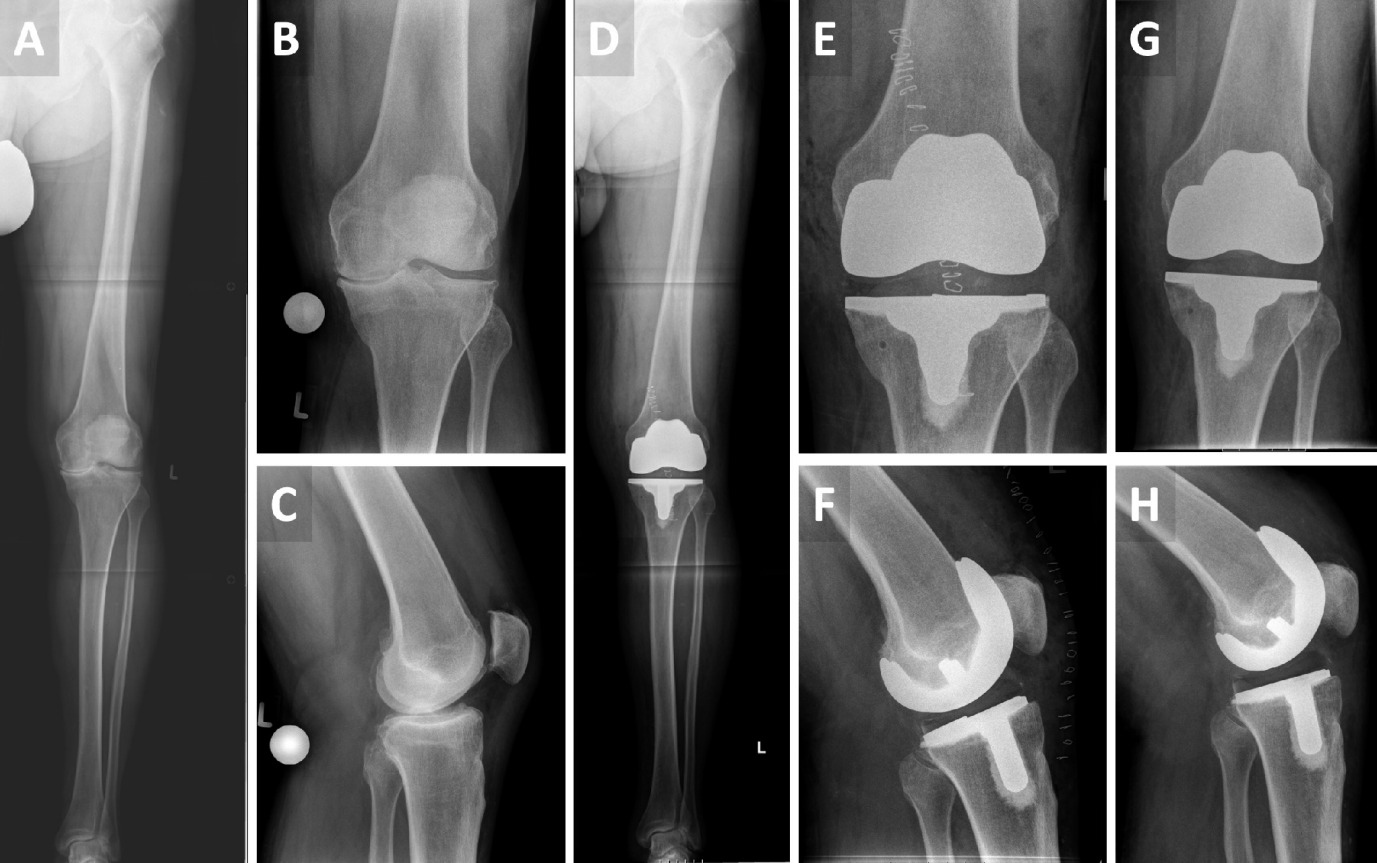
Exemplary radiographic imaging **A**,** B**,** C**: preoperative radiographs **D**,** E**,** F**: immediate postoperative radiographs **G**,** H**: radiographs at last follow-up

Surgeries were performed by three senior arthroplasty surgeons at a tertiary care academic hospital employing a dedicated enhanced recovery after surgery (ERAS) protocol. A medial parapatellar approach was routinely used, and the surgery was performed with the provided, patient-specific cutting guides aiming for neutral mechanical alignment starting at the femur. Ligamentous stability was tested, and, if necessary, the resection level was adjusted at the tibia using the cutting guides. Tibial and femoral implants were cemented. There was no usage of tourniquets or wound drainage. In the absence of contraindications, patients received 1 g of intra-articular Tranexamic acid and periarticular and subcutaneous infiltration with local anesthetics (Ropivacaine). Full-weight bearing was initiated on the day of surgery. Figure 2 shows exemplary radiographs taken before (A, B, C) and after surgery (D, E, F), along with imaging from the most recent follow-up (G, H).

### Follow-up period and methodology

Patients were followed up prospectively and received a letter for self-reporting their outcomes five years after the surgery. The mean follow-up duration was 5.9 ± 0.8 years (range 5–7.4 years). Data collection included patient satisfaction, pain levels using the Visual Analogue Scale (VAS), current weight, the Oxford Knee Score (OKS), and the Forgotten Joint Score for the knee (FJS-knee). A five-year follow-up was defined as “mid-term” according to Ahmad et al. [19] and is far above the period of 6–12 months where patients usually achieve their “plateau” of functional outcome [20].

### Outcome measures

Patient-reported outcome measures (PROMs) included a standardized written questionnaire assessing their satisfaction [21]. Besides, they were asked if they would do the same operation again. Besides, pain levels at rest and during exercise were evaluated using the Visual Analogue Scale for pain (VAS). Furthermore, patients were asked about their current weight which was then compared to the weight at surgery. Finally, the Oxford Knee Score [22] and the Forgotten Joint Score [23] were assessed. In case patients reported complications, they were scheduled for a consultation for further evaluation.

### Statistical analysis

Descriptive statistics were used to summarize the demographic and clinical characteristics of the patients. Outcomes were reported as means with standard deviations (SD) and ranges.

## Results

One hundred sixteen patients who received a CIM TKA (ConforMIS iTotal CR) were followed up with a mean duration of 5.9 years (range, 5–7.4).

**Fig. 3:**
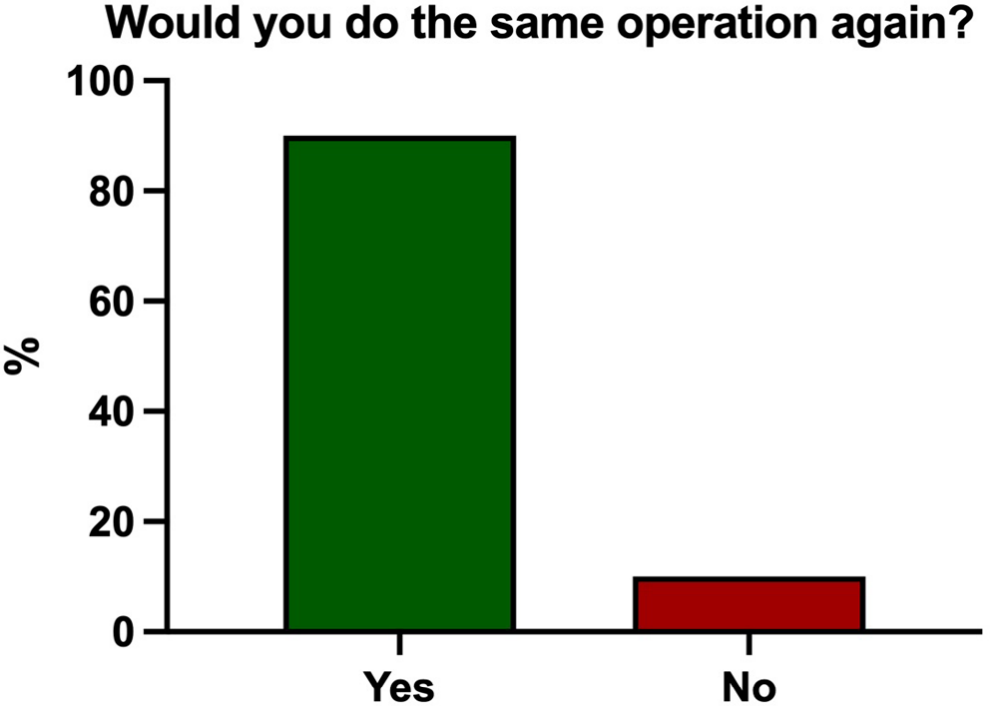
90% of the patients said they would do the same operation again (*n* = 115)

**Fig. 4:**
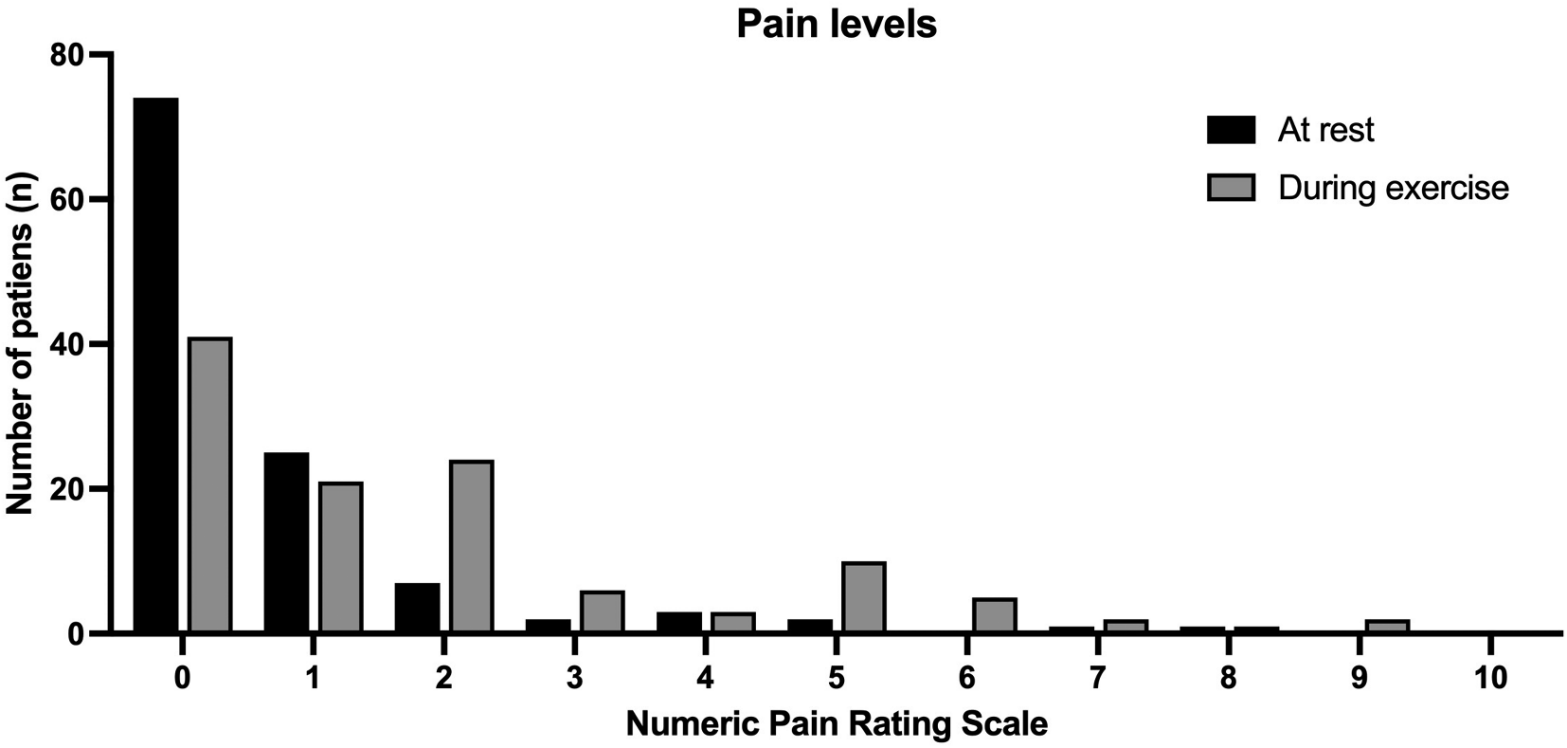
Numeric pain rating scale at rest and during exercise (*n* = 115)

**Fig. 5:**
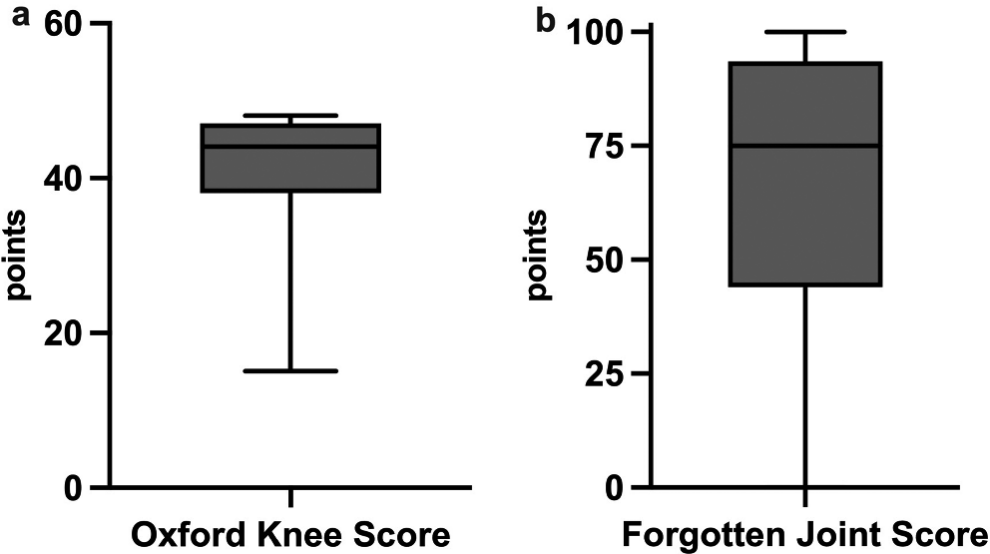
Oxford Knee Score **(a)** and Forgotten Joint Score for the knee **(b)** The mean Oxford Knee Score was 41 ± 8. The mean Forgotten Knee Joint Score was 67 ± 30

90% of patients said they would do the same operation again (Fig. 3). 94% of patients were either satisfied (23%) or very satisfied (71%) with their outcome; further satisfaction rates and functional outcomes are depicted in Table 2. Pain levels at rest and during exercise are shown in Fig. 4. 53% of patients lost weight after the operation (Table 3). The mean Oxford Knee Score was 41 points (range, 15–48) (Fig. 5a), and the mean Forgotten Knee Joint Score was 67 points (range, 4–100) (Fig. 5b).

**Table 2:** Questionnaires evaluating patient satisfaction

What is your overall satisfaction with the operation?	Number of patients (*n*)	(%)
Very satisfied	82	71%
satisfied	27	23%
unsatisfied	3	3%
Unsatisfied	4	3%
**How satisfied are you with** **the pain reduction?**	**Number of Patients (n)**	**(%)**
Very satisfied	84	72%
satisfied	25	22%
unsatisfied	4	3%
Unsatisfied	3	3%
**How satisfied are you concerning the improvement of your ability to perform housework and gardening?**	**Number of Patients (n)**	**(%)**
Very satisfied	71	61%
satisfied	34	29%
unsatisfied	5	4%
Unsatisfied	6	5%
**How satisfied are you concerning the improvement of your ability to perform free time activities?**	**Number of Patients (n)**	**(%)**
Very satisfied	66	57%
satisfied	36	31%
unsatisfied	9	8%
Unsatisfied	4	3%

Patients answered four questions regarding their satisfaction using a 4-point Likert scale. Results are shown as absolute and relative values

**Table 3:** Postoperative weight dynamics

Dynamic	Number of patients (*n*)	(%)
Increase in weight (↑)	36	33%
TSteady weight (→)	15	14%
Weight loss (↓)	58	53%

Patient weight before the operation was compared to the reported weight five years after surgery. Results are shown in absolute and relative values

A total of three revisions were recorded: One patient was revised due to MCL insufficiency, one due to patellar instability and one due to persistently decreased range of motion. No infections or implant loosening were recorded.

## Discussion

The most important finding of the present study is that CIM TKA with mechanical alignment can achieve high levels of patient satisfaction even at mid-term with more than five years of follow-up. In fact, 94% of patients were either satisfied or very satisfied, and 90% would elect do undergo the same operation again.

Satisfaction levels in our study are located at the upper quartile compared with those reported for OTS TKA. The mean OKS for OTS implants ranges between 16.4 and 43.6, depending on the study [24–26]. Likewise, our results align with those reported for the FJS-knee [27]. In a recent study, Gousopoulos et al. reported a 94% satisfaction rate two years after CIM TKA using the Symbios CR implant [17]. With an OKS of 39.6 points and a FJS-knee of 69.0 points, their results align with those in the present study (OKS = 41, FJS-knee = 67). Interestingly, the mean FJS-knee of 67 points, achieved by our patients, is almost equal to that of a healthy and younger population of 2017 US citizens, reaching 66.8 points on average [28].

White et al. reported a higher rate of postoperative stiffness, which eventually required manipulation in a group of patients receiving CIM TKA using ConforMIS compared to OTS implants at two years follow-up [29]. Even though there was no personal consultation to assess the Range of Motion after five years, there were no complaints of postoperative stiffness recorded in our cohort. None of the 164 patients received manipulation under anesthesia at our institution postoperatively. This aligns with a later study from Wheatley et al. that confirmed no higher rate of manipulation after CIM TKA [30].

After proving the efficacy and safety of CIM TKA at mid-terms, one pending question remains unanswered: Does CIM TKA provide a better outcome than OTS TKA? While several RCTs have compared patient-specific to standard instrumentation [31, 32], so far no RCTs comparing CIM to OTS implants have been published. Hence, there are only cohort studies, mostly retrospective, available. In such a study, Wendelspiess et al. found no difference in outcomes after 12 months comparing the ConforMIS iTotal with the Attune^®^ CR mobile-bearing implant (DePuy Synthes, Raynham, MA, US) [33]. In contrast, Reimann et al. found higher levels of patient satisfaction after two to three years by comparing the ConforMIS iTotal with the Triathlon^®^ Total Knee system (Stryker, Kalamazoo, Michigan, USA). Interestingly, in patients with bilateral TKA, where one was CIM, and one was OTS, patients tended to prefer the CIM side [34].

It has to be noted that the available CIM knee implants differ greatly in terms of configuration, alignment strategy, and overall philosophy. The Origin^®^ implant (Symbios, Yverdon les Bains, Switzerland) aims for constitutional alignment within predetermined limits, uses a single-block polyethylene Inlay, and provides individual patellofemoral configuration. In contrast, the ConforMIS^®^ iTotalCR implant (ConforMIS Inc., Boston, Massachusetts, USA) aims to restore neutral mechanical alignment and uses two polyethylene Inlays [35]. Both systems offer a posterior stabilized version. Importantly, the notion of “fully restoring” knee kinematics with CIM implants might not be entirely accurate. First, it has to be noted, that there are different definitions (hip-knee-talus vs. hip-knee-calcaneus) of mechanical alignment [36]. Besides, when the anterior cruciate ligament (ACL) or both the ACL and posterior cruciate ligament (PCL) are resected and a cruciate-retaining (CR) or posterior-stabilized (PS) insert is used, paradoxical anterior gliding can still occur despite “restoring” bony anatomy. CIM implants, such as those by Symbios or ConforMIS, do not typically feature a medial-pivot (MP) design that could potentially address this issue.

This study has several limitations: Scores and patient satisfaction were only evaluated postoperatively and therefore change scores could not be calculated. Besides, there was no personal consultation of the patients, hence no assessment of ROM by a medical professional, and no follow-up imaging was performed. Furthermore, there was no specification of pain location in case of persistence. Knowing the exact location of pain, for instance anterior knee pain, might bring further clarity into the need for personalized patellofemoral joints since this is not provided by the implant studied. A strength of the study is the extended follow-up of at least five years, with an acceptable percentage of patients lost to follow-up (29.3%).

## Conclusion

CIM TKA using the ConforMIS^®^ iTotal CR implant can achieve excellent results with 93% of patients being satisfied or very satisfied at mid-term follow-up of five years. Prospective randomized and patient-blinded trials comparing off-the-shelf (OTS) TKAs with CIM implants are necessary to evaluate whether these implants are superior or not.

## Data Availability

The raw data supporting the conclusions of this article will be made available by the authors on reasonable request.

## Abbreviations

TKA: Total Knee Arthroplasty
CIM: Customized Individually Made
VAS: Visual Analogue Scale
OKS: Oxford Knee Score
FJS-knee: Forgotten Joint Score for the knee
PROMs: Patient-Reported Outcome Measures
OTS: Off The Shelf
ERAS: Enhanced Recovery After Surgery
